# Identity of Two Sympatric Species of *Orius* (Hemiptera: Heteroptera: Anthocoridae)

**DOI:** 10.1673/031.010.18901

**Published:** 2010-11-01

**Authors:** Jeffrey P. Shapiro, Paul D. Shirk, Karen Kelley, Tamera M. Lewis, David R. Horton

**Affiliations:** ^1^ Center for Medical, Agricultural, and Veterinary Entomology, Agricultural Research Service, U.S. Department of Agriculture, Gainesville, FL 32608; ^2^ University of Florida, Interdisciplinary Center for Biotechnology Research, Gainesville, FL 32610; ^3^ Yakima Agricultural Research Laboratory, U.S. Department of Agriculture, Wapato, WA 98951-9651

**Keywords:** 18s DNA, genitalia, minute pirate bugs, sexual competition, yolk protein ELISA

## Abstract

The minute pirate bugs, *Orius insidiosus* (Say) and *Orius pumilio* (Champion) (Hemiptera: Heteroptera: Anthocoridae), are closely related species known to be sympatric in north Florida. Here, male and female genitalia, DNA sequences, and the effects of within- and between-species pairings on egg production and egg development were examined to develop a better understanding of the relationship between these two species. Interspecific matings between the two species did not result in viable progeny. Although there were gross similarities in the morphology of the male parameres (external genitalia) between the two species, the cone in *O. pumilio* was much broader with a greater spiral twist and the flagellum was longer than in *O. insidiosus*. Correspondingly, there were differences in the morphology of the copulatory tubes of the females of the two species. In *O. insidiosus*, the organ was somewhat longer than in *O. pumilio* and oriented parallel to the abdominal midline, while the copulatory tube in *O. pumilio* tilted slightly towards the midline. Additionally, the copulatory tube for *O. pumilio* included a sclerotized basal mound that was not present in *O. insidiosus*. These morphological differences suggest that successful copulation between these species could be difficult. In contrast to conspecific matings, interspecific matings resulted in few or no eggs laid over a period of two weeks and no viable progeny. Comparison of the 18S ribosomal gene ITS-1 sequences between the two species demonstrated only 91% homology. When yolk protein contents were examined to determine whether reproductive physiology had shifted to full egg production, interspecifically mated females contained amounts of yolk protein comparable to that in fed, but unmated females; this was less than 10% of the yolk protein previously found in fed and conspecifically mated females. These findings together confirm that *O. insidiosus* and *O. pumilio* are indeed two separate species.

## Introduction

The minute pirate bugs (Hemiptera: Heteroptera: Anthocoridae) comprise 500 to 600 species of Heteroptera worldwide. These insects are important as natural members of the predatory fauna (Lattin 2000) and as biological control agents in many agroecosystems (Stiling and Cornelissen 2005; van Lenteren et al. 2006; Zehnder et al. 2007 ). Two species of pirate bugs, *Orius insidiosus* (Say) and *Orius pumilio* (Champion), were found coexisting on an organic farm in north central Florida (Shapiro et al. 2009). Reports of both species have been made from Central America, Mexico, Jamaica, Cuba, and the United States (Champion 1900; Kelton 1963; Herring 1966; Carpintero 2002; Shapiro et al. 2009). In the continental U.S., the range of *O. insidiosus* encompasses the areas east of the Rocky Mountains, north to Canada, and south to Florida. It is the most widespread species of *Orius* in the western hemisphere. The coexistence of *O. insidiosus* and *O. pumilio* in the United States has only been observed in Florida, where the northerly limit of *O. pumilio* appears to be Alachua County, Florida. The two species were found together during the spring on the flowers of two umbelliferous plants, false Queen Anne's lace (*Ammi majus* L.) and Queen Anne's lace (*Daucus carota* L.) (Apiales: Umbelliferae [Apiaceae]), where both pirate bugs apparently preyed on Florida flower thrips, *Frankliniella bispinosa* (Morgan) (Thysanoptera: Thripidae). Throughout the four weeks of study, the demographics for the two species differed markedly. The *O. insidiosus* population was heavily weighted toward males, with a sex ratio of 2.7 males: females (Shapiro et al. 2009), and the *O. insidiosus* outnumbered the *O. pumilio* population by 3.6-fold. These observations have led to questions about ecological niches, interspecific competition, and the potential for interspecific mating competition and sexual conflict between the two species.

Fundamental to understanding these interactions is a clear grasp of the taxonomic relationships between *O. insidiosus* and *O. pumilio*. Genital morphologies have conventionally served as key taxonomic characters for the Heteroptera in general and for the Anthocoridae in particular. The size, shape, and orientation of the copulatory tube in the female (Carayon 1961; Pééricart 1972; Yasunaga 1997; Bu and Zheng 2001; Silveira et al. 2003) and the shape of the paramere in the male (Ribaut 1923; Herring 1966; Pééricart 1972) are used as diagnostic characters in identifying *Orius* species. In addition to genitalic morphologies, the appearance, coloration, and morphometrics of antennal segments, head, thorax, scent glands, and wings have all contributed to the taxonomic identity of genera and species (e.g. Herring 1966, 1976).

However, these conventional identifying characters do not assure certainty in discriminating interacting field populations of other anthocorids. For example, *Anthocoris antevolens* White (Heteroptera: Anthocoridae) has a broad range in North America. Comparison of specimens from various regions results in a high degree of morphological variability and uncertainty in differentiating them from the closely related species *Anthocoris musculus* (Say) (Horton and Lewis 2005; Horton et al. 2005, 2007, 2008). Because the morphologically different populations of *A. antevolens* are overlapping and sympatric with *A. musculus* populations, potential inter- and intraspecific matings among all these groups could occur. Indeed, laboratory trials showed that insemination can occur in certain heterospecific pairings (Horton et al, 2005, 2008). Thus, the identity of these species was re-examined based on morphologies of various body parts and genitalia, and included examining the relatedness of mitochondrial DNA sequences. These investigators demonstrated that there was significant uncertainty in identifying any of the populations as a species especially when utilizing the current taxonomic keys.

With their widespread distributions, *Orius* species may also prove to be adapted and reproductively isolated as local and regional populations along with closely related species. In Japan, *Orius* species have been shown to be adapted latitudinally in their diapause characteristics perhaps reflecting their genetic relatedness and adaptations that have led to speciation (Musolin et al. 2004; Musolin and Ito 2008; Shimizu and Kawasaki 2001). Whether other aspects of physiological and behavioral adaptation in anthocorids, both to changes in the physical environment and to pressures exerted by closely related species, result in divergence of populations and ultimately to speciation remains to be discovered.

In order to more reliably assess the relationship between these two species of *Orius*, the morphologies of male and female genitalia, the abilities to cross-mate and produce subsequent egg development, and comparisons of genomic sequence have been examined here. While both species had previously been reported to occur in Florida (Blatchley 1926; Kelton 1963; Herring 1966), only limited observations have been made for the two species in areas where they coexist; these observations included limited monitoring of populations over a period of weeks during the daylight hours in the spring of 2008 (Shapiro et al. 2009; unpublished data, 2009) in the flower heads of two species of Umbelliferae (*A. majus* and *D. carota*). A more detailed morphological and genetic description of the relationship between these two species in Florida may contribute to a discussion of interspecific convergence (or divergence) in genitalic morphology and gene sequences, and the potential significance of inter- and conspecific mating competition, sexual conflict, and competition for resources such as prey and pollen.

## Materials and Methods

### Insect colonies

A colony of *O. insidiosus* was established from a field population collected in Alachua County, Florida in 2008 (Shapiro et al. 2009). A colony of *O. pumilio* was established in 2002 from insects collected in Bronson, Levy County, Florida, 18 miles southwest of the site from where the *O. insidiosus* population was collected (Shapiro and Ferkovich 2009). Subcolonies of 24 h egg collections from each species were set up from primary colonies, and insects were allowed to develop for 13–14 d before use.

### Interspecific matings

From sub-colonies of each species, unsexed 5^th^ instar nymphs were individually isolated by species and allowed to molt to adults in microtiter plate wells. Adults were anaesthetized in place with CO2 0–2 d following the adult molt, sexed, and mating was initiated at day 0 in conspecific and heterospecific crosses. Each replicate consisted of 10 males and 10 females in a Petri dish (50 ×× 9-mm) covered with a nylonscreened (23-mm diameter, 0.2-mm sieve size) tight-fit lid, containing 0.2 g shredded parchment paper and 0.75 g of 5% sucrose Hydrocapsules (ARS Inc.). Each group was moved into a 0.6-L Mason jar for oviposition at day 7. Jars contained 1 green bean, 3.5 g buckwheat hulls, 1.0 g Hydrocapsules®®, and 0.1 g *Ephestia kuehniella* eggs. Every 2–3 days until day 21, eggs were counted, beans were replaced, and 0.15 ml *E. kuehniella* eggs were added. At day 18, surviving adults were counted and their species identities were noted. Oviposition was terminated at day 21. For yolk protein ELISA analysis, adult mating groups were set up and mated as above at day 0 and collected at day 6, survivors were counted, and 3 females were collected for each replicate and stored at -80°°C in a 2-ml microcentrifuge tube.

### Scanning electron microscopy

Terminal segments or isolated parameres were dissected from males anaesthetized with CO2 or preserved in 100% ethanol and then transferred into 20% KOH for 2 d at room temperature for clearing. Segments and parameres were transferred from KOH through H_2_O, 25%, 50%, and 100% ethanol in succession over <1 hr, and stored in 100% ethanol. Segments or parameres were dried from ethanol and mounted with carbon adhesive tabs on aluminum stubs. Specimens were Au/Pd sputter-coated (Denton DeskII sputter coater, Denton Vacuum, www.dentonvacuum.com) and then examined with an S-4000 FE-SEM microscope (Hitachi, www.hitachi-hta.com). Digital images were acquired and analyzed with Quartz PCI v8 software (Quartz Imaging Corp., www.qrtz.com). Ten specimens of each species were examined from multiple perspectives and magnifications.

### Light microscopy

The copulatory tubes of adult females were examined in seven specimens of *O. pumilio* and six specimens of *O. insidiosus* from the Gainesville laboratory cultures. In addition, the copulatory tubes of field collected adult females from 13 specimens of *O. insidiosus* collected from eight states in the eastern, central, and western U.S., and of two specimens of *O. pumilio* from southern Florida were examined. Examination of insects from outside of the Gainesville geographic area was done to confirm that copulatory tubes in the Gainesville specimens were representative. The insects from the Gainesville laboratory colonies were preserved in alcohol while field-collected specimens were either dried or freshly killed. The distal half of the abdomen was dissected from each female, soaked in a 10% KOH solution for approximately 4 hr (preserved specimens only) and then transferred to a drop of water on a microscope slide. Abdominal segment VII, bearing the copulatory tube, was removed with the use of microtools. The segment was positioned with the interior side up and covered with a glass slip. Water was drawn out with a paper tissue as needed to flatten the segment. The copulatory tube was photographed at 200x using a digital camera attached to the microscope, and measured using digital software.

### DNA/PCR analyses

The 18S ribosomal gene internal transcribed spacer 1 (ITS-1) has been used previously to assess the phylogenetic relationship among various *Onus* species including *O. insidiosus* and *Orius tristicolor* (White) from North America (Honda et al. 1998). Genomic DNA sequence data for ITS-1 were derived from specimens of *O. insidiosus* and *O. pumilio* collected from our laboratory cultures and from *O. tristicolor* collected from butterfly bush, *Buddleja* sp., growing in Wapato, Washington for comparison with previously published sequences from anthocorid species. Genomic DNAs of 50 pooled adult *O. insidiosus, O. pumilio*, or *O. tristicolor* were isolated using the Wizard Genomic DNA Isolation System (Promega, www.promega.com) according to the manufacturer's protocol for animal tissues. Direct PCR for the ITS-1 and flanking regions were amplified from the genomic DNAs as template using *Taq* PCR kit (New England Biolabs, www.neb.com) with the forward primer, 5′?-ACCGCCCGCGCTACTACCGAT -3′?, and reverse primer, 5′?-TGTTCATGTGTCCTGCAGTTCACA-3′? (Integrated DNA Technologies, www.idtdna.com), as identified by Muraji et al. (2004). The amplification program was 94°° C for 3 min with 40 cycles of 92°° C for 40 s, 58°° C for 40 s, and 72°° C for 40 followed by 72°° C for 4 min. The PCR products were cloned into pGEM-T Easy (Promega) and sequenced on contract using ABI 3130 DNA sequencing at Interdisciplinary Center for Biotechnology Research (University of Florida). A total of six sequences were examined for each species to establish nucleotide identities.

The nucleotide sequences for ITS-1 reported from anthocorid species were aligned using ClustalW as a component of Mac Vector 7.2.3 software (MacVector, Inc., www.macvector.com). The phylogenetic and molecular evolutionary analyses were conducted with PAUP** v 4.0b10 (Swofford 2002) and Mega version 4 (Tamura et al., 2007). Alignments were subjected to analysis with PAUP** Maximum Parsimony and trees were established as rooted using *Cimex lectularius* Linnaeus as the outgroup with a 50% majority rule consensus and a bootstrap of 1000 replicates. The same sequence alignment was subjected to analysis with Mega4 Neighbor-Joining, Minimum Evolution and Maximum Parsimony-Close-Neighbor Interchange, and all phylograms were established as rooted using *C. lectularius* as the outgroup with a 50% majority rule consensus and a bootstrap of 1000 replicates.

**Table 1.**
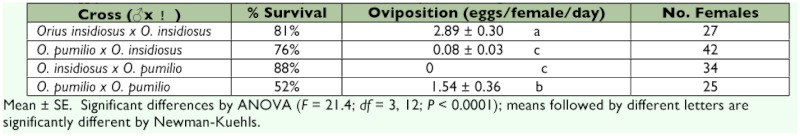
Egg production and mortality in conspecific and heterospecific matings.

### Yolk protein quantification

A monoclonal antibody-based ELISA was used as described (Shapiro and Ferkovich 2002), with minor modification (Shapiro and Shirk in press), to quantify the amount of yolk protein in adult female *O. insidiosus* and *O. pumilio*.

## Results

### Interspecific matings

The outcome of interspecies matings were examined to determine whether hybrid progeny could be produced. Conspecific crosses between *O. insidiosus* or *O. pumilio* adults resulted in large numbers of eggs (857 and 424 eggs, respectively; Table 1) and successful production of progeny as would be expected. When placed together in heterospecific crosses, the males of both species attempted mating with the females of the other species. In contrast to the conspecific crosses, heterospecific crosses between the two species resulted in few eggs (37 eggs for *O. pumilio* ♂? ×× *O. insidiosus* ♀?) or no eggs (*O. insidiosus* ♂? ×× *O. pumilio* ♀?). From the 37 eggs laid by the *O. pumilio* ♂? ♀? *O. insidiosus* ♀? cross only 3 F1 offspring were recovered, but they did not produce offspring themselves. During the course of the experiment, male mortality averaged 22% while female mortality was generally lower (18%).

### Morphology of the male paramere

**Figure 1.**
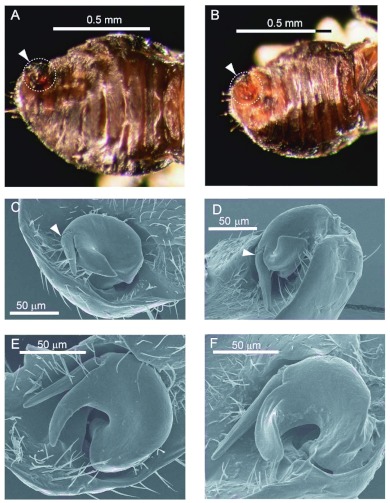
The location and morphology of parameres on the dorsal abdomen of male *Orius insidiosus* (A, C, E) and *O. pumilio* (B, D, F). Panels A and B: Reflected light views showing the location of the parameres on the tergite of the ninth abdominal segment (within the dotted circle). Panels C, D, E, and F: SEM views showing the position of the parameres within a cuticular depression. The position of the flagellar hinge is marked with white arrowheads in Panels C and D. High quality figures are available online.

As in other Anthocoridae, the left parameres of *O. insidiosus* and *O. pumilio* are dorsally situated on the ninth abdominal segment (Figure 1A, B). During copulation, the abdomen of the male of either species is extended around and beneath (ventral to) the female. Subsequently the paramere rotates, pivoting counterclockwise around the axis of its embedded root (Figure 1C, E) and comes into contact ventrally with the copulatory tube of the female. Under lower magnifications, differences in the morphology of the excised parameres from *O. insidiosus* and *O. pumilio* are not discernable although parameres from *O. pumilio* appear more robust than those from *O. insidiosus* when viewed laterally (edgewise).

**Figure 2.**
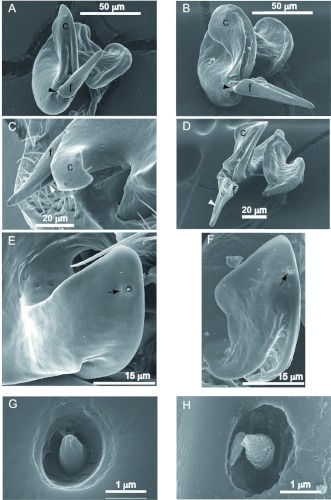
SEM micrographs showing the morphology of cones and sensilla of *Orius insidiosus* (A, C, E, G) and *O. pumilio* (B, D, F, H). The position of the hinge between the cone (c) and flagellum (f) is marked by a black arrowhead in panels A and B. The groove on the inner surface of the flagellum is marked by a white arrowhead in panels C and D. The position of the sensillum on the outer surface of the cone is marked by an arrow in panels E and F. High quality figures are available online.

When viewed with the SEM, the images of the parameres (Figures 1–2) revealed structural features not readily visible at the lower magnification. In both species, the flagellum of the paramere was relatively short and somewhat blade- or spear-shaped (Figures 1 C–F, 2 A–B). Morphological differences between the species were noted that were consistent with drawings of Herring (1966, Figs. 14, 15). However, caution must be exercised when comparing the parameres because differences in the angular view of the parameres can cause visual distortions of the structures. The cone of the *O. insidiosus* paramere (Figure 2A) was shorter than that in *O. pumilio* (Figure 2B), did not spiral in as extensive an arc, and was more spear-shaped compared to that of *O. pumilio*. The cone of *O. pumilio* had a more spatulate shape and spiraled further out of the plane (Figure 2B). A groove (““canal”” in Ribaut 1923 or ““furrow”” in Pééricart 1972) was noticeable on the cone of the paramere, extending from at least the junction of the cone and flagellum towards the apical tip of the cone (Figure 2A–D). According to Pééricart (1972), the furrow extends to the ventral side of the cone (not shown). The flagellum in the paramere of *O. pumilio* (Figure 1F) was also relatively longer than that of *O. insidiosus* (Figure 1E).

In both species, there was a noticeable pliability in the dissected paramere at the junction of the cone and flagellum; i.e. the flagellum appears to be flexibly hinged at this junction (arrowheads in Figures 1 C, D; 2 A, B). Both species also exhibited a pronounced furrow or groove in the flagellum (Figure 2B, C), shown clearly extending along the length of the flagellum for *O. pumilio* (Figure 2D). This flagellar groove has not previously been reported in any species of *Orius*. Finally, a single pit sensillum (arrows, Figure 2E, F) was apparent on the outer surface close to the tip of the cone in both species. At the highest magnifications, the structure of the sensillum of both species revealed a peg within the pit (Figure 2G, H). The function of this structure is not known.

### Morphology of the female copulatory tube

**Figure 3.**
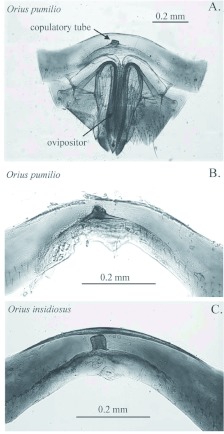
Copulatory tubes of female *Orius pumilio* (A, B) and *O. insidiosus* (C). Panel A: Location of copulatory tube relative to location of ovipositor (shown here for *O. pumilio*; laboratory culture); 100x. B: Copulatory tube in *O. pumilio* (laboratory culture); 200x. C: Copulatory tube in *O. insidiosus* (laboratory culture); 200x. High quality figures are available online.

The copulatory tube in both species was observed as a short, sclerotized, and thickwalled cylinder slightly offset from the longitudinal midline of the sternite (Figure 3A–C). For both species, the sperm pouch located at the terminal end of the copulatory tube was destroyed during the dissections and is not shown. A thin-walled apical section usually observed in the copulatory tube of females in this genus (Carayon 1958, Pééricart 1972) was not visible in either species. The copulatory tubes of the two species differed in length, orientation and presence of a basal sclerotized mound. The copulatory tube in *O. pumilio* was composed of a cylindrical tube about 25 µµm long (range in Gainesville specimens: 15–37 µµm; range in two south Florida specimens: 15–26 µµm) that was embedded in or continuous with a sclerotized basal mound (Figure 3B). The width of the cylindrical tube is generally 1.3 to 2.0 times its length (range in all dissected specimens: 30–48 µµm). The tube was tilted toward the longitudinal midline of the abdomen, although in some specimens or dissections it could be nearly erect. The basal mound and cylindrical portion together had the appearance of a truncated cone (Figure 3B). In *O. insidiosus*, the copulatory tube was about 35 µµm in length (range in Gainesville specimens: 31–48 µµm), and was approximately as wide as it was long (Figure 3C). The copulatory tube was generally parallel to the longitudinal midline of the abdomen, but in some specimens or dissections was oriented slightly away from the midline. In this species, there was no sclerotized mound surrounding the base of the cylindrical tube. In both species, the general appearance of the copulatory tube in the Gainesville specimens was similar to appearance in specimens from outside of the Gainesville area.

### ITS-1 conservation

**Figure 4.**
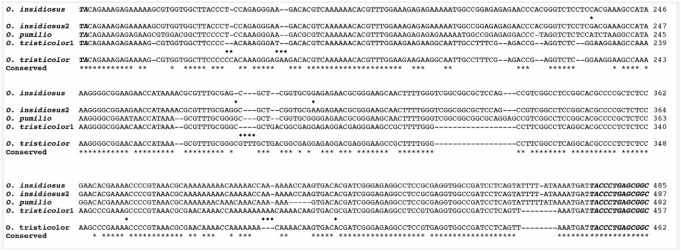
Sequence alignments of 18S rDNA from *Orius insidiosus, O. pumilio*, and *O. tristicolor*. The region shown covers the ITS-1 from each sequence and the more conserved sequence regions of 18S rDNA PCR product were not shown. The *O. insidiosus*2, *O. pumilio*, and *O. tristicolor* 1 ITS-1 sequences produced here were aligned with published sequences for *O. insidiosus* (AF061187) and *O. tristicolor* (AF061374). The *bold/italic* bases at the 5′? and 3′? ends represent rDNA sequences. The (——) indicates a missing base, a (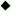) between the sequences for a species designates a mismatched base with the published sequence, and the (**) below the sequences designates a conserved base present in all aligned sequences. High quality figures are available online.

The ITS-1 PCR primers produced a product of approximately 600bp that included the ITS-1 and flanking rDNA sequences from genomic DNA of each of the 3 species tested, *O. insidiosus, O. pumilio*, and *O. tristicolor*. Alignment with previously published sequences for *O. insidiosus* and *O. tristicolor* (Honda et al. 1998) showed only 3 base pairs were different over the length of the ITS-1 between the *O. insidiosus* published sequence and the one produced here (Figure 4). The sequence for *O. tristicolor* showed 14 bp different between the two sequences. Because the published sequences were derived from samples collected in Arizona (Honda et al. 1998), these differences may indicate regional divergence in the ITS-1 sequence for these two species. When compared with *O. insidiosus*, the ITS-1 sequence from *O. pumilio* had 472 of 521 (91%) shared base identities, while there were only 433 of 516 (84%) shared base identities with *O. tristicolor*. Similarly, *O. insidiosus* and *O. tristicolor* had 432 of 521 (83%) shared base identities.

**Figure 5.**
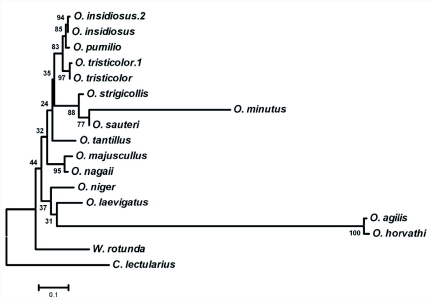
A phylogram of *Orius* species based on similarities between 18S ITS-1 sequences from various species of the Anthocoridae and Cimicidae. The ITS-1 DNA sequences from published and derived sequences were aligned against a 710 base segment of *Wollastoniella rotunda* Yasunaga and Miyamoto (Hemiptera: Heteroptera: Anthocoridae) ITS-1 (AF061375) with ClustalW. The phylogram was generated from the alignment with Mega4 using Neighbor-Joining rooted with *Cimex lectularius* Linnaeus (Hemiptera: Heteroptera: Cimicidae) as the outgroup, and with a bootstrap of 1000. The published ITS-1 sequences were from *Cimex lectularius* (EU 126968), *O. agilis* (Flor) (EF487296), *O. horvathi* (Reuter) (EF487299), *O. insidiosus* (AF061187), *O. laevigatas* (Fieber) (AF061366), *O. majusculus* (Reuter) (AF061367), *O. minutus* (Linnaeus) (AF061368), *O. nagaii* Yasunaga (AF061369), *O. niger* (Wolff) (AF061370), *O. sauteri* (Poppius) (AF061371), *O. strigicollis* (Poppius) (AF061372), *O. tantillus* (Motschulsky) (AF061373), and *O. tristicolor* (AF061374). The *O. insidiosus*-2, *O. pumilio*, and *O. tristicolor*-1 ITS-1 sequences were produced here. The bootstrap confidence levels are shown on the branches and the bar denotes substitutions per base pair. High quality figures are available online.

To assess the relative phylogenetic relationships among the various *Orius* species from which ITS-1 sequences have been reported, molecular evolutionary analyses were conducted to resolve the alignment of the ITS-1 sequences for anthocorids (Honda et al. 1998) with those identified here using multiple approaches. Four different phylograms were generated utilizing different selective criteria, and consistently the *Orius* species found in Japan segregated as a clade with *O. minutus, O. sauteri*, and *O. strigicollis* grouped closely together as previously observed (Honda et al. 1998). The other major clade observed in all four phylograms included the three species from North and Central America, i.e. *O. insidiosus, O. pumilio*, and *O. tristicolor*, as a related group with *O. insidiosus* and *O. pumilio* the more closely related (Figure 5).

### Yolk protein quantification in interspecific matings

Conspecific and heterospecific matings between *O. insidiosus* and *O. pumilio* were done to assess the physiological impact of potential interspecific interactions. When conspecific matings were maintained for 27 d prior to ELISA and dissections, no significant differences in yolk protein (22 µµg/female in *O. insidiosus vs*. 17 µµg/female in *O. pumilio*) or egg contents (10.2 eggs/female in *O. insidiosus* vs. 10.7 eggs/female in *O. pumilio*) were found (Table 2).

When heterospecific crosses were conducted for 6 d, the conspecific control matings resulted in approximately 25% more yolk protein per female in *O. insidiosus* than in *O. pumilio* (24.9 *vs*. 19.3 µµg/female, respectively) (Table 3). However, when the heterospecifically mated females were examined, the yolk protein content of the females of both species was at least 10-fold less than that of the conspecifically mated females (1.3 µµg/female for *O. pumilio* ×× *O. insidiosus* and 1.8 µµg/female for *O. insidiosus* ×× *O. pumilio*) (Table 3). These yolk protein contents were similar to those observed in fed, unmated *O. pumilio* females (Shapiro and Shirk, in press).

## Discussion

The closely-related minute pirate bugs, *O. insidiosus* and *O. pumilio*, both occur in Central America, Mexico, Jamaica, Cuba, and Florida (Champion 1900; Kelton 1963; Herring 1966; Shapiro et al. 2009) apparently in sympatry. Recently these two species have been found coexisting naturally in the flowers of Umbelliferae (Apiaceae) where they fed on an abundance of Florida flower thrips, *F. bispinosa* (Shapiro et al. 2009). These floral feeding stations evidently provided an environmental oasis where both species found plentiful food and an increased opportunity for mating. However, the skewed abundance of male *O. insidiosus* and the dominant numbers of *O. insidiosus* over *O. pumilio* at this site (Shapiro et al. 2009) suggest that the population dynamics of the two species are perturbed in this setting. Because these two species are similar in external appearance (Herring 1966), we were interested in assessing whether these are true, reproductively-isolated species that have been shown to coexist in sympatry, or whether their sympatry could potentially lead to hybrid progeny or disrupted mating patterns as both species apparently compete for one ecological niche. However, the findings presented here confirm *O. insidiosus* and *O. pumilio* are clearly distinct species. This conclusion was based on detailed comparisons of male and female genital morphology, by examination of 18s rDNA ITS-1 DNA sequences, by the lack of significant egg production following interspecific mating, and by the inability to effect a change in female reproductive physiology (egg maturation) following interspecific mating (Table 3).

Genitalia typically serve as key taxonomic characters in the Heteroptera and are used extensively in the Anthocoridae to identify species as well as for developing higher level groupings of species (Carayon 1972; Pééricart 1972; Yasunaga 1997; Bu and Zheng 2001). The size, shape, and orientation of the copulatory tube in the female (Carayon 1961; Pééricart 1972; Yasunaga 1997; Bu and Zheng 2001; Silveira et al. 2003) and the shape of the paramere in the male (Ribaut 1923; Herring 1966; Pééricart 1972) are used as diagnostic characters in identifying *Orius* species. An illustration of the copulatory tube in *O. insidiosus* can be found in Silveira et al. (2003), but one in Carayon (1972) is incorrectly labeled as a copulatory tube from *O. insidiosus*. The micrographs of the copulatory tube from *O. pumilio* presented here are the first descriptions published for this species. The copulatory tube was somewhat longer in *O. insidiosus* than in *O. pumilio*, and the copulatory tube of *O. insidiosus* was oriented parallel to the abdominal midline while that of *O. pumilio* was slightly tilted toward the midline. The copulatory tube of *O. pumilio* also had a broad, sclerotized basal mound that was not present in *O. insidiosus*.

**Table 2.**
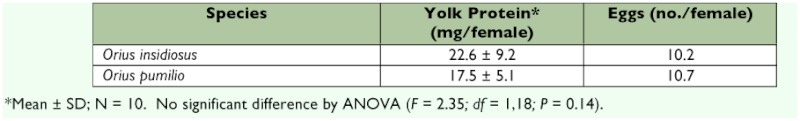
Yolk protein content of fed and mated females.

**Table 3.**
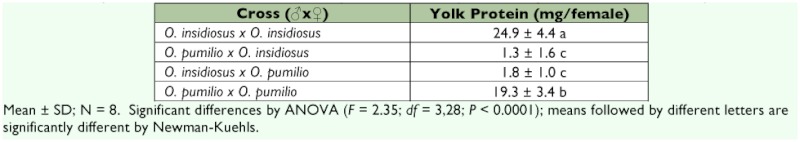
Yolk protein contents of females from conspecific and heterospecific matings of *Orius insidiosus* and *O. pumilio*.

Males are often identified to species by the morphology of the left paramere (Pééricart 1972, Yasunaga 1997). The paramere functions during copulation by apparently guiding the soft tissue of the phallus through the groove between flagellum and cone into the copulatory tube of the female (Pééricart 1972). The right paramere is vestigial or nonexistent in the Anthocoridae (Schuh and Slater 1995). The parameres of *O. pumilio* and *O. insidiosus* are illustrated in Kelton (1963) and Herring (1966). However, these drawings fail to show substantial morphological detail. To acquire greater detail for comparing the parameres in *O. insidiosus* and *O. pumilio*, specimens were examined with scanning electron microscopy. Differences in the shapes of the parameres were especially distinct at these higher magnifications. The less prominent, spear-shaped cone in *O. insidiosus* contrasted with the spatulate, elongated cone in *O. pumilio*. In both of these species, the flagellum was reduced in length and curvature from those described for other *Orius* species from North America (Herring 1966), although that of *O. pumilio* was longer than the flagellum of *O. insidiosus*. A novel unanticipated structural feature of the parameres in these two species was the presence of a single sensillum distinctly visible on the outer curvature of the cone of the parameres. The function of the sensillum was not examined here.

Several molecular markers have previously been used to establish relatedness among species or geographic variants of anthocorids, among them sequences of the 18s rDNA internal transcribed spacer 1 (ITS-1). Previously, ITS-1 was used to examine the relationships between *Orius* species found in Japan (Honda et al. 1998; Muraji et al. 2004). The ITS-1 sequences of *O. insidiosus* and *O. pumilio* shared only 91% homology while there was only 1bp difference between the ITS-1 sequence of *O. insidiosus* from Florida (present results) and those previously reported for specimens originating in Arizona (Honda et al. 1998). Neither *O. insidiosus* nor *O. pumilio* ITS-1 sequences shared more than 84% homology with the sequence of *O. tristicolor*, the other major North American species. Regardless of the method used to establish the phylograms, the phylogenetic analyses based on the ITS-1 sequences for the anthocorids consistently associated the three *Orius* species from North America in one clade and the major group of *Orius* species from Japan in another clade. The grouping of the *Orius* species from Japan is consistent with previous reports (Honda et al. 1998). These comparisons further support the taxonomic groupings based on morphological features and phylogenetically place *O. insidiosus* and *O. pumilio* as closely related species.

The interspecific differences in shapes of parameres and copulatory tubes described above may not provide mechanical fits adequate for interspecific matings. To test whether successful interspecific mating is possible and can result in hybrid progeny, *O. insidiosus* and *O. pumilio* were cross-mated. Although no eggs were produced when *O. pumilio* females were paired with *O. insidiosus* males, a few eggs were laid by *O. insidiosus* females paired with *O. pumilio* males. However, only three of these eggs hatched and produced nymphs that reached the adult stage. These studies substantiated the reproductive isolation between these two species, but do raise the possibility that some interspecific mating and egg production may occur in nature, although the potential for viable hybrid progeny seems minimal. Furthermore, even if copulation and sperm transfer were successful in interspecific matings, those matings did not result in increased yolk protein synthesis and accumulation much greater than the levels observed in fed-but-unmated females (Shapiro and Shirk, in press). Although a few oviposited eggs were obtained from the *O. pumilio* ×× *O. insidiosus* matings, the small number of eggs could have been oviposited by very few females. Adult female *O. pumilio* require both feeding and mating to stimulate the female to achieve maximum yolk protein production and develop eggs; if the female is only fed, but left unmated, a low level of yolk protein is produced yet no eggs develop (Shapiro and Shirk, in press). The level of yolk protein observed in the females of both species from the interspecific matings was consistent with fed, but unmated females in the interspecific matings. This suggests that either the interspecific males were unsuccessful in copulation and sperm transfer and thus ineffective at mating, or that the act of copulation and the transfer of sperm were not sufficient to stimulate a switch in reproductive physiology.

Horton et al. (2005) examined mating behavior and success between three populations of *Anthocoris antevolens* White (Hemiptera: Heteroptera: Anthocoridae), two of which were sympatric. As with the present species of *Orius*, males of *A. antevolens* had variable success in mating with females from other populations. While one cross between individuals of the two sympatric populations showed a low frequency of success in insemination, the reciprocal cross showed no success. In the case of *A. antevolens*, the reproductive isolation was interpreted as evidence that the species actually comprises a complex of an unknown number of cryptic species (Horton et al. 2005, 2008). These findings punctuated the historical uncertainty concerning taxonomic relationships between *A. musculus* (Say) and *A. antevolens*, particularly highlighting their status as separate species (Horton et al. 2008). Perhaps this quandary could be extended to *Orius* as well, for example in questioning whether the broad distribution of *O. insidiosus* might in fact result in broad diversification of cryptic species. Diapause is one example of a character that exhibits such diversity within the genus *Orius* and within species, and latitudinal changes commonly result in corresponding change in the inducibility of diapause (Musolin et al. 2004; Musolin and Ito 2008; Shimizu and Kawasaki 2001). Whether other aspects of physiological and behavioral adaptation in anthocorids, both to changes in the physical environment and to pressures exerted by closely related species, result in divergence of populations and speciation remains to be discovered.

These morphological, molecular, genetic, and ecological observations support a close phylogenetic relationship between the two species, and leave room for speculation regarding species divergence. It was concluded that these two species are more closely related to each other than either one is to *O. tristicolor*, or to Old World species. As often noted, accelerated divergence and speciation may be expected in sympatric species when sexual conflict occurs, especially between males of one species and females of the other species (Arnqvist et al. 2000, Panhuis et al. 2001). Further behavioral studies may clarify such interactions in these *Orius* species, and a study on the effects of interspecific sex ratios on reproduction is ongoing.

## Abbreviations

**ELISA**,: Enzyme-linked immunoserological assay;
**ITS-I**,: Internal transcribed spacer I;
**PCR**,: polymerase chain reaction;
**rDNA**,: ribosomal DNA;
**SEM**,: scanning electron microscopy
